# RNAseq analysis of treatment-dependent signaling changes during inflammation in a mouse cutaneous wound healing model

**DOI:** 10.1186/s12864-021-08083-2

**Published:** 2021-11-25

**Authors:** Georges St. Laurent, Ian Toma, Bernd Seilheimer, Konstantin Cesnulevicius, Myron Schultz, Michael Tackett, Jianhua Zhou, Maxim Ri, Dmitry Shtokalo, Denis Antonets, Tisha Jepson, Timothy A. McCaffrey

**Affiliations:** 1 The St. Laurent Institute, Vancouver, WA USA; 2 SeqLL, Inc., Woburn, MA USA; 3 Department of Medicine, Division of Genomic Medicine, The George Washington University Medical Center, 2300 Eye St, Washington D.C, 20037 USA; 4 Heel GmbH, Baden-Baden, Germany; 5 AcademGene, LLC, Novosibirsk, Russia; 6 A.P. Ershov Institute of Informatics Systems, Novosibirsk, Russia

**Keywords:** Inflammation, Transcriptome, RNA sequencing, Traumeel, Diclofenac, Cyclooxygenase, Lipoxygenase, Phospholipase

## Abstract

**Background:**

Despite proven therapeutic effects in inflammatory conditions, the specific mechanisms of phytochemical therapies are not well understood. The transcriptome effects of Traumeel (Tr14), a multicomponent natural product, and diclofenac, a non-selective cyclooxygenase (COX) inhibitor, were compared in a mouse cutaneous wound healing model to identify both known and novel pathways for the anti-inflammatory effect of plant-derived natural products.

**Methods:**

Skin samples from abraded mice were analyzed by single-molecule, amplification-free RNAseq transcript profiling at 7 points between 12 and 192 h after injury. Immediately after injury, the wounds were treated with either diclofenac, Tr14, or placebo control (*n* = 7 per group/time). RNAseq levels were compared between treatment and control at each time point using a systems biology approach.

**Results:**

At early time points (12–36 h), both control and Tr14-treated wounds showed marked increase in the inducible COX2 enzyme mRNA, while diclofenac-treated wounds did not. Tr14, in contrast, modulated lipoxygenase transcripts, especially ALOX12/15, and phospholipases involved in arachidonate metabolism. Notably, Tr14 modulated a group of cell-type specific markers, including the T cell receptor, that could be explained by an overarching effect on the type of cells that were recruited into the wound tissue.

**Conclusions:**

Tr14 and diclofenac had very different effects on the COX/LOX synthetic pathway after cutaneous wounding. Tr14 allowed normal autoinduction of COX2 mRNA, but suppressed mRNA levels for key enzymes in the leukotriene synthetic pathway. Tr14 appeared to have a broad ‘phytocellular’ effect on the wound transcriptome by altering the balance of cell types present in the wound.

**Supplementary Information:**

The online version contains supplementary material available at 10.1186/s12864-021-08083-2.

## Background

The complex physiological events that comprise the acute wound healing process may be central to understanding the biological mechanisms of chronic disease. Tissue injury and healing mechanisms share remarkable similarities in different organs [1]. Even in cancer, the importance of tissue remodeling has led authors to describe tumors as having a component of “wounds that do not heal” [2]. Transcriptome-based studies have revealed inflammatory molecular signatures in many diseases, and precisely defined many of the inflammatory events that dominate the early stages of tissue repair [3]. Detailed analysis of wound dynamics over time is beginning to redefine chronic disease states. For example, the discovery of the resolvins and their actions within the inflammation system have suggested that injury “resolution” is a defining event between “acute” and “chronic” inflammation [4, 5].

The present study interrogates a high-resolution map of the mouse transcriptome during wound healing to define changes resulting from therapeutic intervention with Traumeel (Tr14), a well-known natural multicomponent anti-inflammatory medicinal product for musculoskeletal conditions. Natural products can have a broad spectrum of important biological effects ranging from the antimitotic and anticancer effects of taxol, purified from the Pacific yew tree bark (*Taxus brevifolia*), to the anti-inflammatory effects of aspirin, a non-steroidal anti-inflammatory drug (NSAID), derived from precursors in willow tree bark (genus *Salix*). Natural products have been used for millennia to inhibit inflammation in various forms, and may target multiple points in the inflammation pathways [6], including the prostaglandin/leukotriene pathways [7, 8]. Prior studies have demonstrated that Tr14 inhibits IL-1β and TNF-α production by resting and activated immune cells in vitro [9], and has antioxidative properties [10]. In clinical studies, Tr14 has shown effects on cytokine levels in exercise-induced muscle injury [11, 12], and demonstrated pain relief in acute ankle sprains [13]. NSAIDs, such as diclofenac, have a fairly well-defined mechanism of action via cyclooxygenase inhibition. However, NSAIDs have diverse secondary effects that might be better understood by characterizing their effects on the transcriptome [14, 15]. We have previously reported the transcriptome-wide analysis of Tr14 therapeutic effects in the mouse wound healing model [3]. The present studies were conducted to compare and explore more specifically the anti-inflammatory effects of Tr14 and diclofenac at the transcriptome level.

## Results

### Global signature of diclofenac versus Tr14

**Fig. 1:**
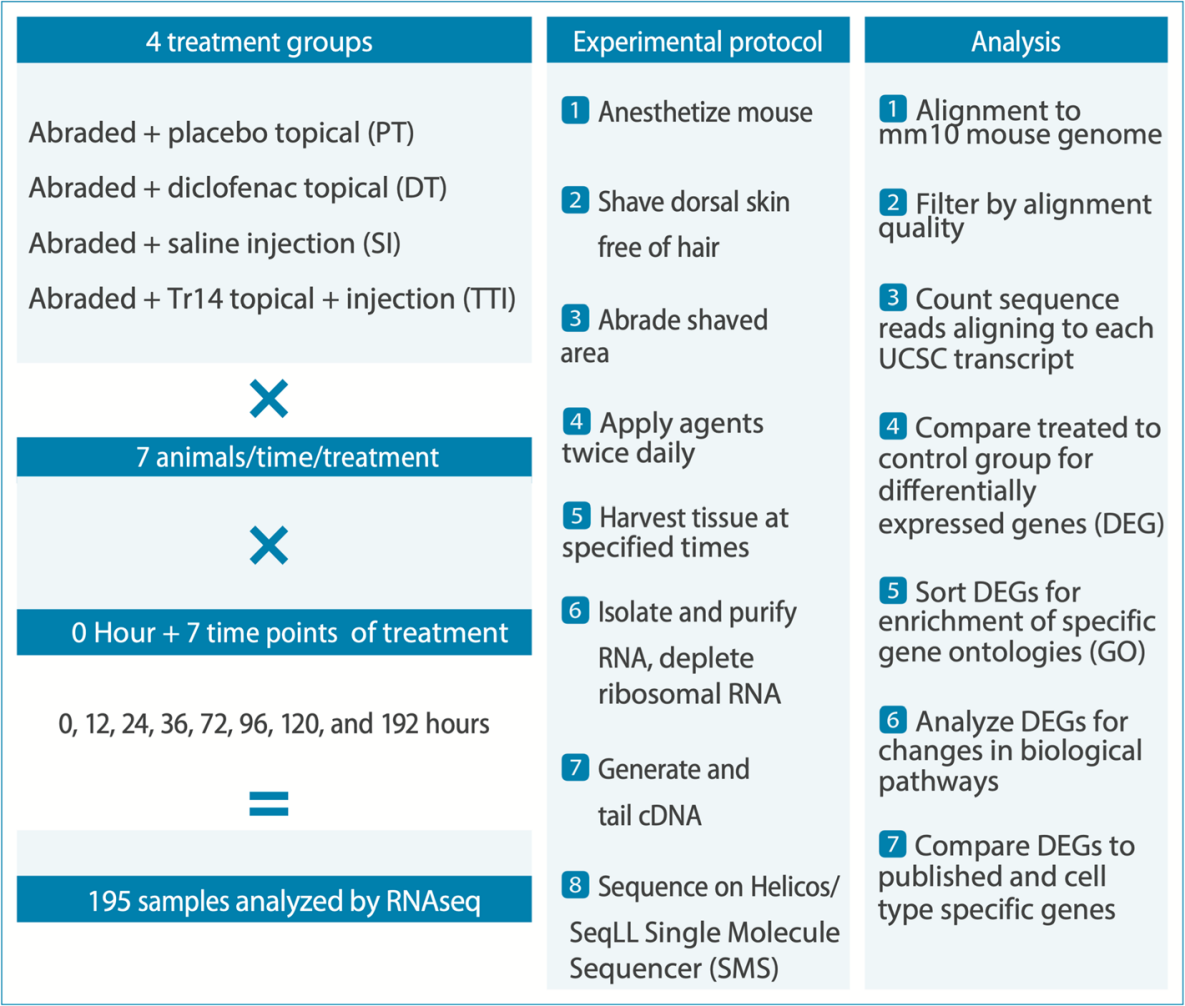
Schematic diagram of study design. The overall study design is summarized schematically to describe the groups, the wounding model, RNAseq analysis, and bioinformatic analysis

**Fig. 2:**
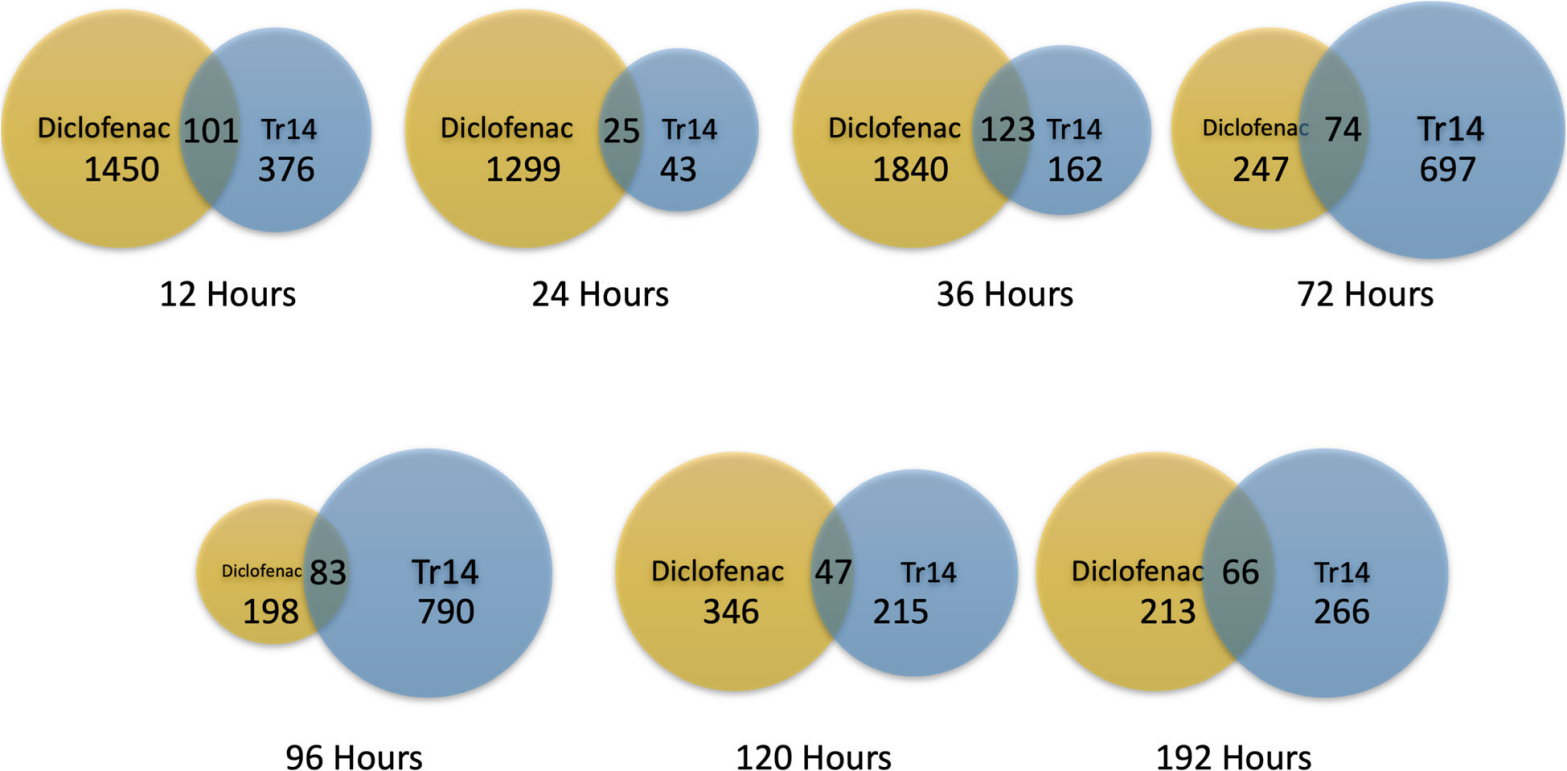
Venn diagrams of overlapping transcripts between diclofenac and Tr14 treatment. RNAseq quantitation (RPK10M) of transcript levels were compared between diclofenac vs control and Tr14 versus control to identify differentially expressed genes (DEGs) for each treatment group (log2 fold-change > + 0.5 with Benjamini-Hochberg adjusted *p*-value < 0.000001–0.001). The number of DEGs is shown for diclofenac (orange) and Tr14 (blue) at each of time points measured. The number of shared transcripts is shown in the overlapping area

The overall similarities and differences between the two types of therapy were evaluated by statistically identifying differentially expressed genes (DEGs) and then organizing the DEGs according to Gene Ontologies to identify any systematic patterns of change as shown schematically in Fig. 1 (all DEGs listed in Suppl. Table 1). In general, diclofenac induced a larger number of expression changes compared to Tr14, when similar statistical filters were applied (Fig. 2). This was mainly observed at the earlier time points of 12–36 h post-injury, where diclofenac altered as much as 10 times as many transcripts as Tr14 (36 h, 1840 vs 162 DEG). By 72–96 h however, Tr14 altered about 3 times more transcripts than diclofenac, with the effects roughly balanced by 96–120 h (Fig. 2). When the specific DEGs between groups were compared, there were 25–123 common transcripts altered by both of the treatments, that reflected roughly 10–50% overlap between the DEGs at any given time point (Fig. 2). That degree of overlap is 5.6 to 30-fold greater than expected by chance, with the odds of such an overlap occurring by chance alone being extremely small, with a range of *p* = 2.2 × 10^− 18^ (24 h) to 8.4 × 10^− 189^ (96 h) by Fisher’s exact test. Thus, the two treatments affected both shared and unique RNA transcripts over time.

### Bioinformatic analysis of diclofenac and Tr14-modulated transcripts

**Fig. 3:**
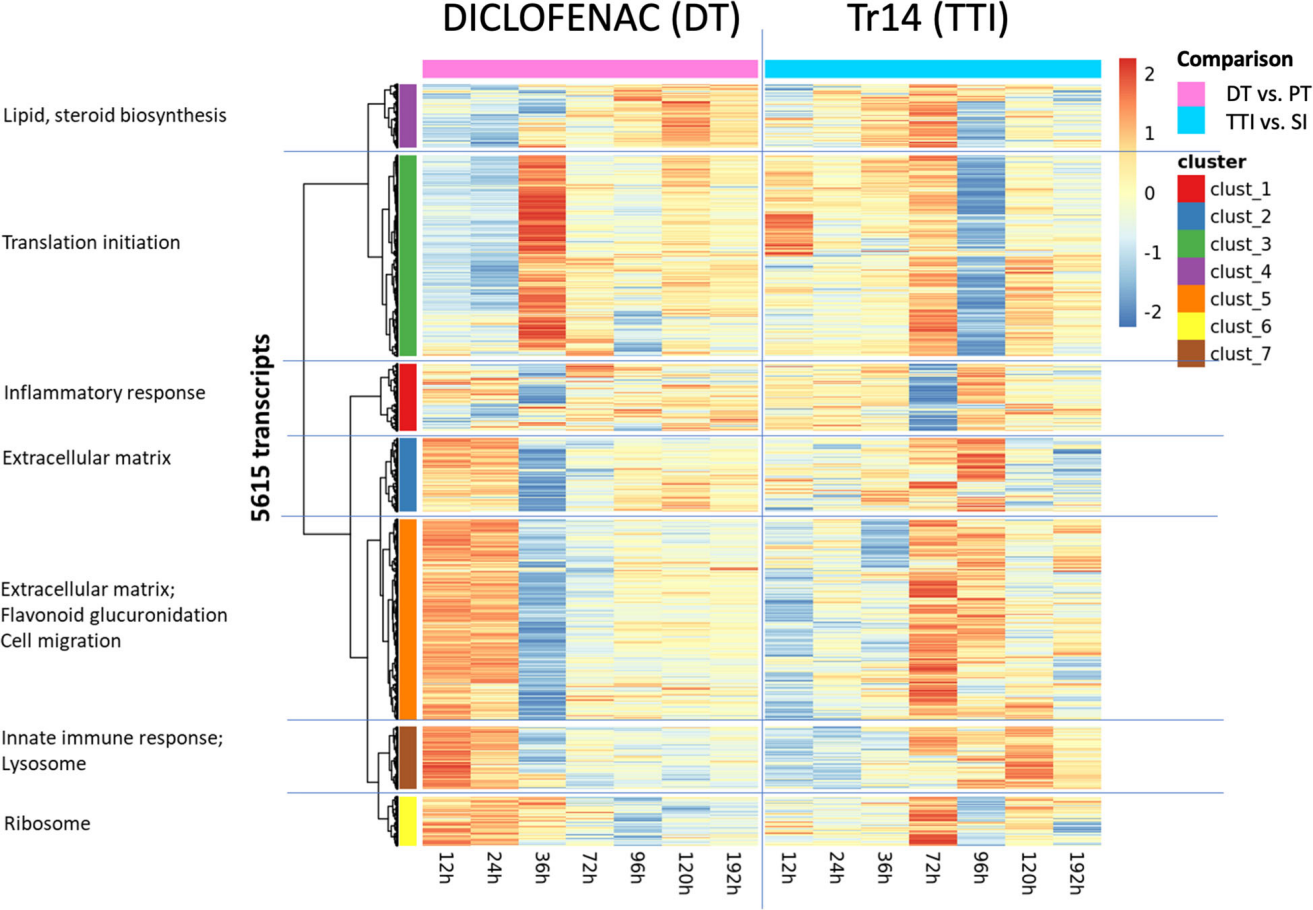
Treatment-dependent gene expression during the wound healing time course. Across all the time points and treatment conditions, 5615 transcripts were differentially expressed (*p* < 0.001). According to the time and treatment conditions, the differential expression levels were scaled by row, and clustered

The types of transcripts affected by each treatment over time were classified into pre-curated gene ontologies (GO) according to their functions. Tr14 induced a clusters of transcripts related to lipid/steroid biosynthesis and translation initiation in the 12–72 h timeframe, that were not observed to increase in diclofenac-treated wounds. Both clusters are presented in Fig. 3 as Cluster 3 (green) and Cluster 4 (purple) respectively. When sorted by their relevant gene ontologies, diclofenac had early effects at 12–24 h on transcripts related extracellular matrix, cell migration, innate immunity, and the ribosome. However, by 36 h, this transitioned into effects on the ribosome and translation with waning effects on extracellular matrix transcripts. In contrast, Tr14 had less striking effects on these pathways in the 12–36 h period, while in the later timeframe of 72 to 192 h, Tr14 altered transcripts related to extracellular matrix, innate immunity, inflammation, and translation (Fig. 3). Thus, while many of the same transcripts and the same systems are altered by diclofenac and Tr14, there was a striking temporal dissociation, with Tr14 showing a delayed effect.

**Fig. 4:**
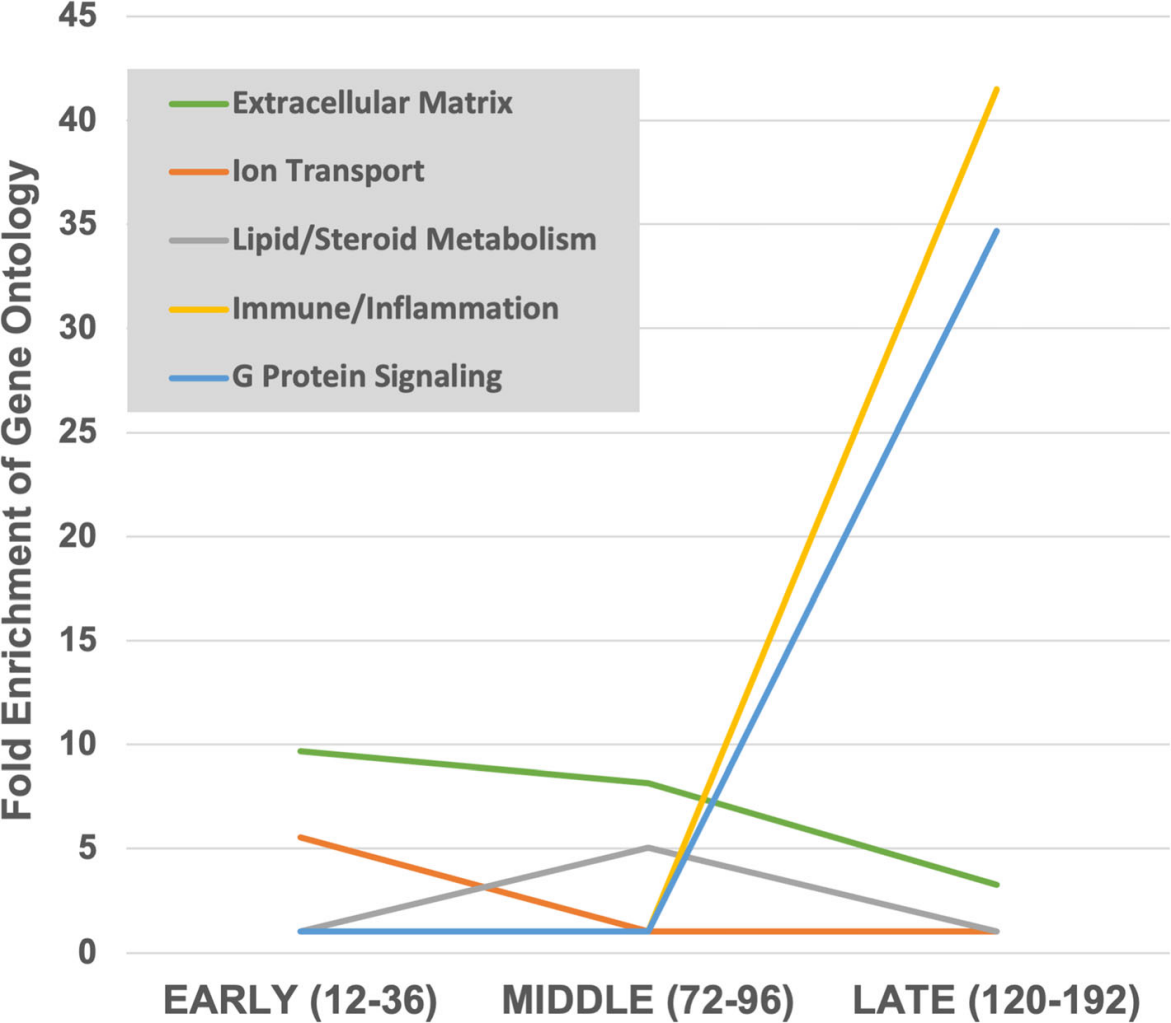
Gene Set Enrichment Analysis (GSEA) of Tr14-modulated transcripts. GSEA of transcripts altered by Tr14 were manually curated into larger ‘SuperClusters’ based on their logical association in biological functions. Tr14 DEGs at each time point were analyzed by GSEA via NCBI DAVID, and then the enriched Gene Ontologies were filtered for false discovery rates (FDR) < 0.05, and manually organized into the functional SuperClusters shown with a computed geometric mean fold change for Tr14 relative to its control

A more detailed examination of the types of transcripts affected by Tr14 points to five ‘SuperClusters’ of transcripts related to immune function and inflammation, extracelular matrix, ion transport, G protein signaling, and lipid/steroid metabolism (Fig. 4). Each of these 5 SuperClusters would be highly relevant to the control of inflammation. Lipid/steroid metabolism, ion transport, and G protein signaling are major pathways that modulate inflammatory signaling and extracelular matrix production. For instance, many of the pro-inflammatory and anti-inflammatory pathways utilize small lipid intermediates that are known to regulate inflammatory and extracelular matrix genes.

### Effects on the eicosanoid pathway transcripts

**Fig. 5:**
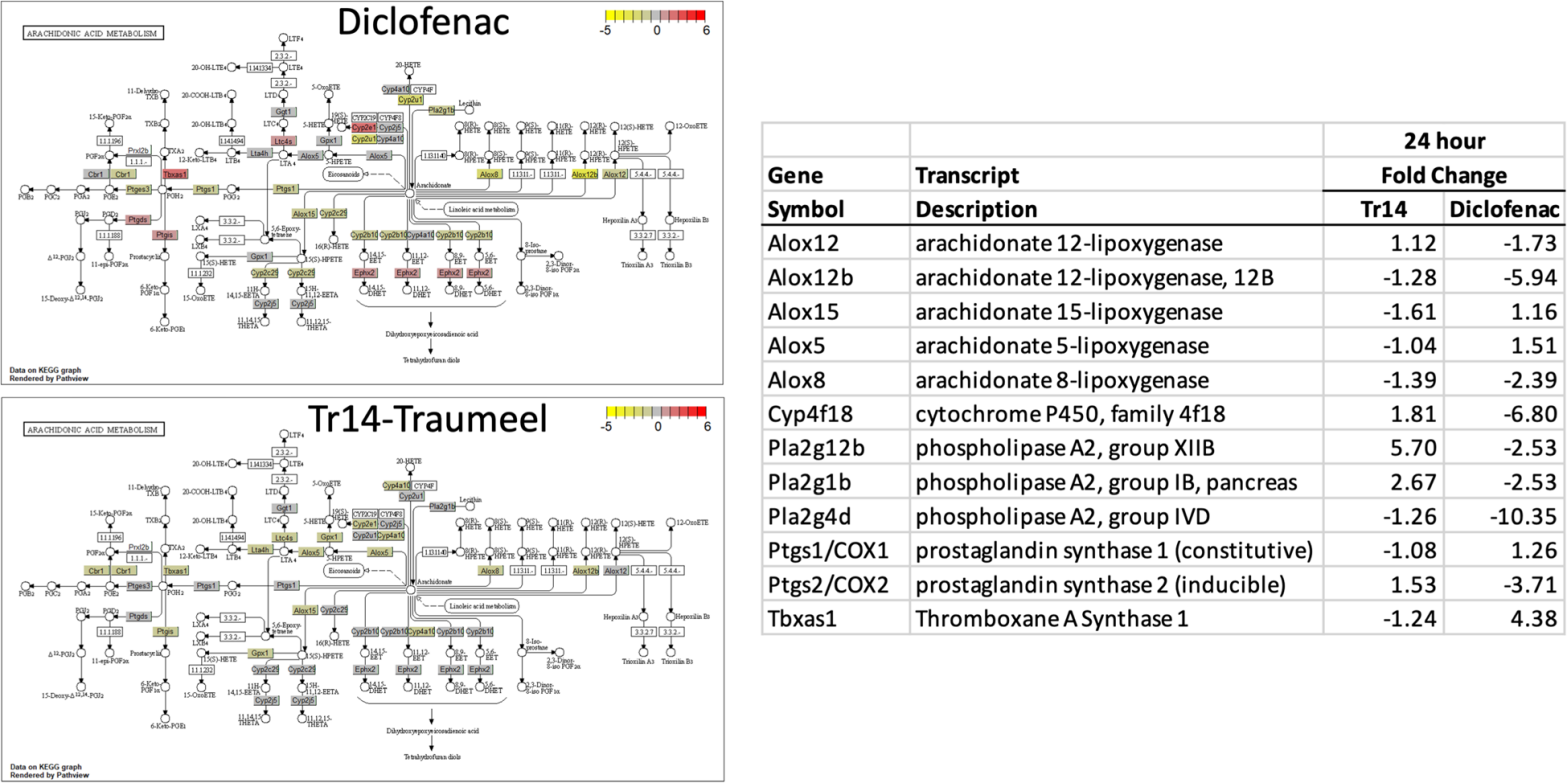
Arachadonic Acid/prostaglandin/leukotriene pathway analysis of treatment-induced changes in the RNA levels at 24 h post-injury. RNA levels were quantified by RNAseq, compared between the diclofenac-treated wounds vs control (upper left panel) and the Tr14-treated wound vs control (lower left panel), and then mapped to the major enzymes in the eicosanoid pathway using KEGG [16]. Red shading indicates the transcript is elevated relative to its control, yellow shading indicates the transcript is reduced relative to control. Proinflammatory pathways are marked with solid line, proresolution pathways marked with dashed line. Numerical fold changes are reported in right panel for selected DEGs in the COX/LOX pathway. All time points are reported in Supplementary Figs. 1-6

The eicosanoid pathway is a well characterized system involving enzymatic production of a variety of small lipid intermediates with pro- and anti-inflammatory effects. The eicosanoid pathway is a known target of NSAIDs, such as diclofenac, and thus, it was interesting to evaluate the effect of these two treatment types on the RNA transcripts in those pathways. Two major pathways of eicosanoid generation involve several catalytic conversions of arachidonic acid into bioactive agents via cyclooxygenases (COX1/2, PGH synthase 1/2, PTGS1/2) and lipoxygenases (LOX) (Fig. 5). In each pathway, several downstream enzymatic steps can significantly alter the biological activity of the eicosanoids in a tissue-specific manner. To examine how these pathways were affected at the mRNA level, the DGE of the enzymes was analyzed in the time series data and compared between treatment groups. At the 24-h time point, for example, diclofenac and Tr14 had very different patterns of changes in COX/LOX-related enzyme mRNAs, showing essentially opposite effects on the modulation of key enzymes such as 5-lipoxygenase (ALOX5), and the phospholipases (Pla2) that are essential for the liberation of arachidonate from the cell membranes. The later time points demonstrate persistent differences in the effects on the COX/LOX pathways (Supplementary Figs. 1–6).

Furthermore, in control wounds, RNAseq detected a strong and well known increase in the inducible PGH synthase 2 mRNA (COX-2, Ptgs1/2) 12–24 h after injury (not shown). As expected from inhibition of the downstream feedback signal, especially PGE2, diclofenac caused an almost 4-fold reduction in COX-2 mRNA expression at early time points, while Tr14, however, did not block COX-2 mRNA induction, which was 1.5 X greater than control levels at 24 h (Fig. 5).

### Pathway analysis of Tr14 versus diclofenac on the pro-inflammatory and pro-resolution eicosanoids

**Fig. 6:**
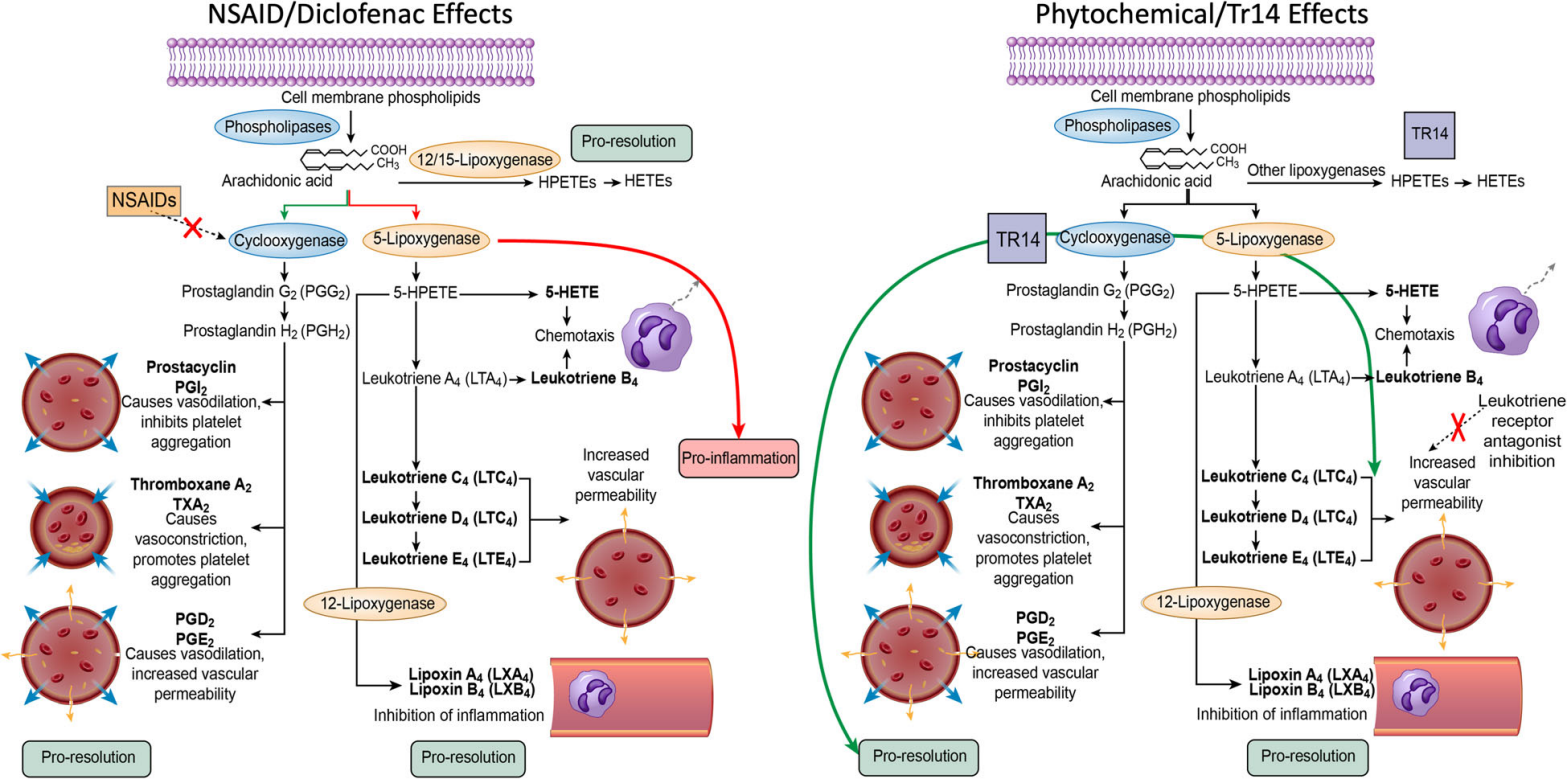
Diclofenac and Tr14 effects in relation to biological pathways of tissue homeostasis and repair. The physiological effects of NSAIDs, such as diclofenac, are shown schematically in the left panel, compared to the putative actions of Tr14 in the right panel. NSAIDS have direct inhibitory on the cyclooxygenase enzymes, which alters numerous downstream eicosanoid actions on vasoconstriction/vasodilation, coagulation, chemotaxis, and inflammation/resolution. Tr14 does not directly inhibit COX enzymes, allowing a more natural wound repair process, but it does alter the mRNA levels for downstream enzymes in the pathway that might favor inflammation resolution

Assembling the above RNA changes in relation to their predicted production of eisosanoids, it is possible to visualize the broad differences between the two agent’s effects on the key pathways in tissue homeostasis and repair. As shown in Fig. 6, NSAIDs such as diclofenac, have relatively specific effects that inhibit the constitutive and inducible COX enzymes, leading to marked reduction in the downstream products PGI2, TBXA2, PGD2, and PGE2. This direct inhibitory effect has the effect of shunting AA into the lipoxygenase pathway, which includes many pro-inflammatory eicosanoids in the leukotriene pathway, but also into the lipoxin pathway which could have beneficial, pro-resolution effects. In contrast, Tr14 has no direct inhibitory effect on COX enzymes, and because downstream mediators such as PGE2 are not impaired, there is a normal and expected increase in COX2/PTGS2 enzyme that is evident at the mRNA level measured here. It is possible that the absence of direct anti-COX activity by Tr14 allows the pro-resolution eicosanoids in that pathway, such as PGI2, PGD2, and PGE2 to exert normal repair functions in proportion to the normal leukotriene and lipoxin pathways.

### Analyzing Tr14 DEGs as markers of migratory cell types in the wound

While manually curating the Tr14 DEGs that were strongly affected early after injury, there was an apparent effect on transcripts that have been previously recognized as markers of particular immune cell types. For example, even at the 12-h time point, Tr14 produced notable effects of up to 17-fold on transcripts associated with T cells such as Dusp14, Fbp1, Ly6a, Ly86, and Sla2 (Table 1). Additionally, several of the Tr14-modulated transcripts were known cell-type markers for the T regulatory subset (Treg), especially FoxP3 and Gata3. Other markers, such as Defb4 (neutrophil) and caldesmon 1 (macrophage) suggest enhanced migration of other immune cell types. While is possible that these transcripts could also be a markers of other resident cell types, collectively they seem to suggest differential migration of immune cells into the wound.

**Table 1:** Immune cell markers modulated by Tr14

Fold change	Adjusted *P*-value	Name	Description	Cell type	Effect/Pathway
1.67	3.31E-08	Cald1	Caldesmon 1	Macrophage	M2 mac infiltration
154.26	3.68E-04	Defb4	Defensin beta 4	Neutrophil	innate immunity
1.28	3.37E-06	Dlg1	Discs Large MAGUK Scaffold 1	T/B/Dendritic	antigen receptor
7.98	3.37E-02	Dusp14	Dual Spec. Phosphatase 14	T cell	neg reg T cells
17.73	6.54E-04	Fbp1	Fructose-Bisphosphatase 1	T cell	Nrf2 pathway
3.32	1.18E-14	Foxp3	Forkhead Box P3	T cell/Treg	consensus marker
4.13	7.67E-01	Gata3	GATA Binding Protein 3	T cell/Treg	FoxP3 system
1.83	2.31E-02	Ly6a	Lymphocyte antigen 6A	Lymphocyte	consensus marker
2.20	3.49E-02	Ly86	Lymphocyte antigen 86	Lymphocyte	consensus marker
1.72	3.13E-02	Mef2c	Myocyte Enhancer Factor 2C	Lym/Myocyte	transcription factor
2.94	5.72E-18	Sla2	Src Like Adaptor 2	T cell	TCR signaling

**Fig. 7:**
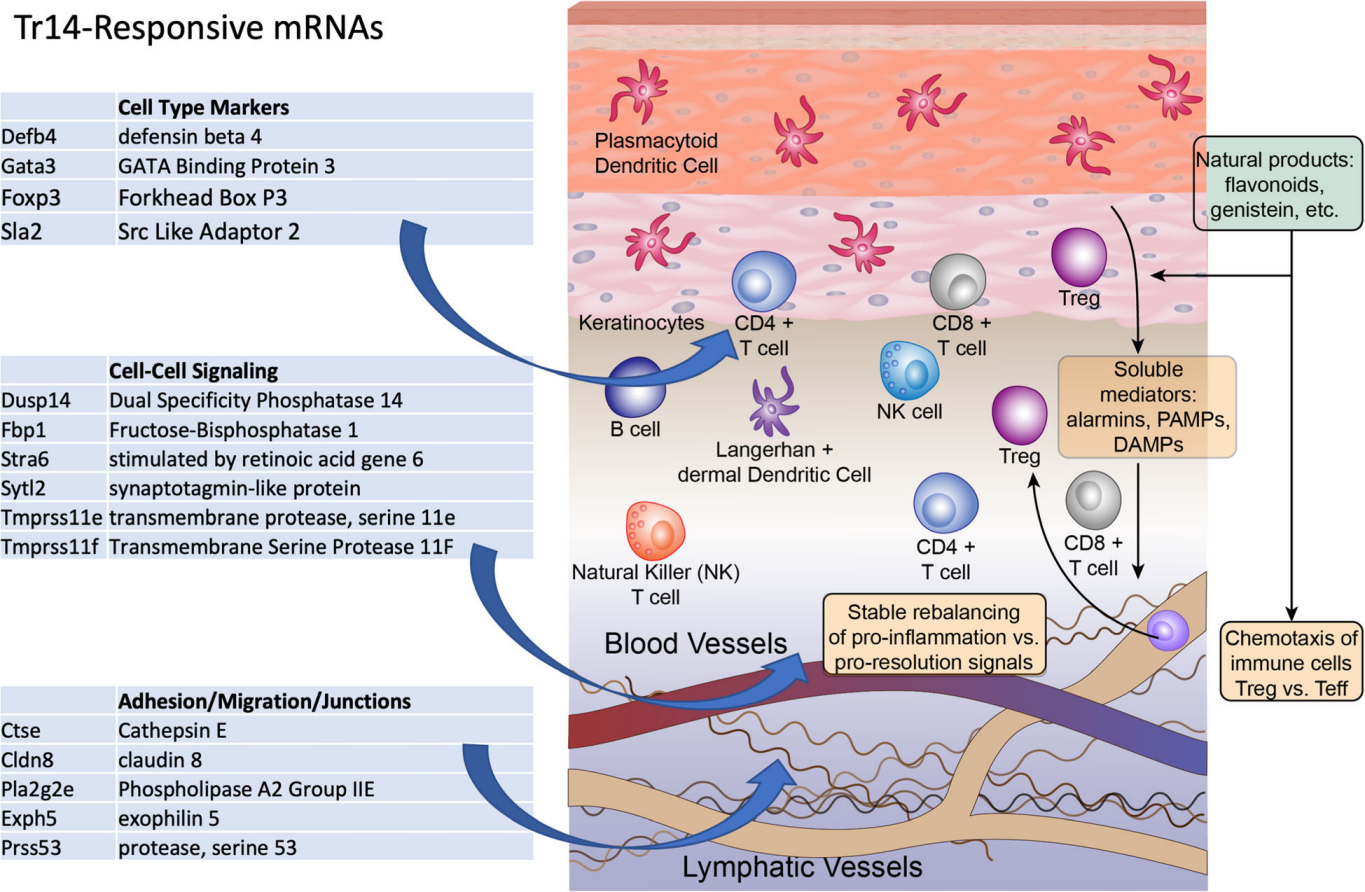
Specific transcripts strongly modulated by Tr14 after injury. Differentially expressed genes (DEGs) modulated by Tr14 are organized as examples of transcripts that would be related to particular cell types present in the wound (i.e. FoxP3), and by transcripts that would modulate Cell-Cell Signaling of those cells (i.e. Dusp14), and their adhesion/migration junctions (i.e. Claudin 8)

These early, strongly modulated transcripts have a common thread of potentially reporting the type of cells that are entering the wound. Related DEGs were also observed that effect cell-cell signaling, adhesion, migration, and intercellular junctions in the wound. As summarized in Fig. 7, these cell type markers may be reporting the types of cells entering the wound, and then other markers are reporting changes in the way that they interact with the wound, especially by altered cell-cell communication. For example, Dusp14 is T cell-associated transcript that negatively regulates T cell receptor (TCR) activation [17]. Other transcripts such as Fbp1 are important in the Nrf2 stress pathway, Stra6 is involved in vitamin A transport in a murine psoriasis model [18], and 2 transmembrane proteases (Tmprss11e/f) are involved in skin barrier function. Other transcripts likely relate to altered intercellular adhesion and migration of immune repair cells, such as cathepsin E, which, as noted, is a neutrophil/T cell factor involved in antigen processing, but is also involved in cell migration. Claudin 8 (Cldn8) is a known epidermal protein involved in tight junctions, and was identified by RNAseq as being relevant to the immune response in atopic dermatitis and psoriasis [19]. Other transcripts are likely related to follicular (Pla2g2e, Prss53) and keratinocyte (Exph5) adhesion and migration, and Pla2g2e has been specifically related to skin disorders such as epidermolysis bullosa and skin fragility [20].

### Comparison of the present dataset to prior studies

The transcriptomic effects of diclofenac have been examined by others, and the present results were compared with the transcriptome analysis of diclofenac treatment of mice by Chung et al. [15]. These authors examined the effect of a single, oral dose of diclofenac (9.5 or 0.95 mg/kg body weight) on the liver transcriptome at 6, 24, and 72 h post-treatment. RNA levels were quantified by microarray, and then 2-way ANOVA was used to identify DEGs. They identified 2 primary pathways that were affected, eicosanoid metabolism and apoptosis, and the present results can confirm some of the changes that they observed. Notably, in the eicosanoid pathway, both studies observed induction of the group 12ª secretory phospholipase A2 isoform 1 (Pla2g12a, similar to Fig. 5 Pla2g), which in the present studies showed an overall 25% induction (*p* < 0.005) across the time course. In the apoptosis pathway, the current studies confirm mild induction of the NFkB inhibitor (Nfkbib, 17% increase, *p* < 0.05), caspase 1 (Casp1, 21% increase, *p* = 0.005) and the p53 tumor suppressor (Trp53, 17% increase, p < 0.05). Likewise, there was modest reduction in lamin B2 RNA levels (Lmnb2, 33% decrease, p < 0.05). Thus, while quite different models, and transcriptome methods, there is some agreement on the types of changes observed after diclofenac treatment.

**Fig. 8:**
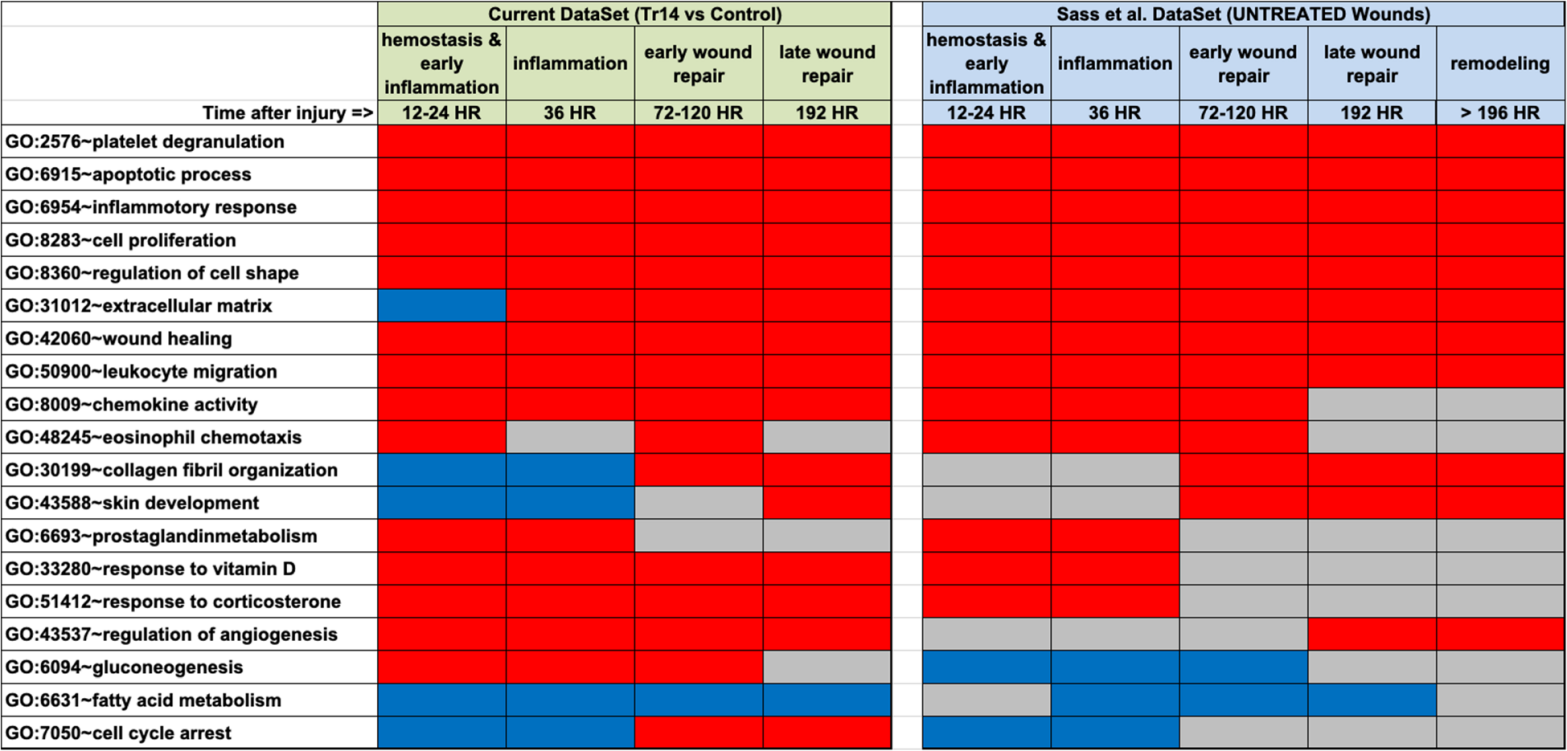
Comparison of the present dataset to prior meta-analysis of wound repair transcript profiling. Enrichment of GO categories reported in the current RNAseq dataset of Tr14 actions (left panels) versus the same GO categories reported by the meta-analysis of non-treated wound repair by Sass et al. (2017), compiled from multiple microarray studies of tissue injury in multiple organs and multiple species. RED indicates increased, BLUE indicates decreased, GREY indicates unchanged

Likewise, Sass et al. [21], conducted an extensive meta-analysis of the transcriptomic changes associated with different injuries to several organ systems (heart, liver, skin, bone, and spinal cord) in 3 different species (rat, mouse, and human). Their aggregated results in the form of gene ontologies affected over time during normal wound repair was compared to a similar analysis of the present data using Tr14-treated wound, and the results are shown in Fig. 8. In general, there is excellent correspondence between the data sets, suggesting that prior microarray studies of normal wound repair identifies the same gene ontologies as are regulated by Tr14 in the current RNAseq studies.

## Discussion

### Transcriptome analysis of murine wound healing

The physiological process of wound healing provides an informative and comprehensive model for approaching the complexity of the tissue pathologies frequently involved in chronic diseases. In many ways, tissues affected by chronic diseases display physiological processes similar to those involved in wound healing, including the potential for inflammation, fibrosis, and scarring. The present studies examine the changes in genome-wide RNA expression patterns resulting from both phytochemical and NSAID therapeutics in the context of a complex physiological wound repair process. This study includes a dataset of over 4 billion sequence reads covering 250 animals, 4 conditions, and 8 time-points, making it a valuable ongoing resource for defining the wound repair process.

### Pathways involved in wound repair

The initial GO enrichment analysis of this data set indicated that Tr14 treatment results in extensive gene expression changes during wound healing, including well known pathways such as TGF-β, cytokine signaling, inflammation, collagen, and enzymes of the extracellular matrix [3]. Interestingly, Tr14-treated mice revealed broad and statistically significant changes in two Gene Ontology groups of great importance to wound healing: Cell Differentiation and Cell Mobility and Migration. These signals may indicate effects upon resident fibroblasts and infiltrating immune cells, which could easily have been overlooked in simpler experimental models, or with methods that are not sensitive enough to detect RNA changes in smaller subsets of cells.

A systems-level analysis of these pathways affected by Tr14 and diclofenac suggested that there were relevant differences in the COX/LOX pathway, which were further explored in the present analysis. While it is common to divide eicosanoid metabolism into the COX and LOX pathways, there is considerable interplay between them, most notably in their shared use of the upstream substrates such as arachidonate acid as shown in Fig. 5. Clinically, it is widely held that the COX pathway produces ‘beneficial’ prostaglandins, such as prostacyclin, which has potent vasodilatory and antiplatelet effects, but also ‘inflammatory’ thromboxanes, such as TXA2, which is vasoconstrictive, platelet aggregating, and pain mediating (Fig. 6). The products of the LOX pathway are principally thought to mediate inflammation, bronchoconstriction, and pain, and thus selective inhibitors of the LOX pathway have been tested in conditions such as asthma and allergies [22].

### The COX pathway

It is well established that injury-induced increases in COX2 mRNA levels are due in large part to NFkB-dependent transcriptional activity [23]. In diclofenac-treated wounds, the COX2 induction is blunted, likely because downstream products, especially PGE2, have been inhibited, and cannot contribute positive feedback to COX2 transcription [24, 25]. Conversely, Tr14 allowed normal induction of the COX2 enzyme, which may have important implications for downstream products, such as the resolvins.

### The LOX pathway

The leukotriene pathway has diverse effects on inflammation and repair, and RNA levels of the enzymes have established relationships to human disease. LTA4 hydrolase mRNA is elevated in conditions such as glomerular inflammation, where immunocytochemistry confirms elevation of the protein product during disease progression [26]. The LTA4 hydrolase promoter contains both positive and negative regulatory elements, but the specific transcription factors have not been identified, although both Nrf2 (NFE2) and Maf sites are present [27]. Likewise, GGT1 has a proximal Nfe2 site, and the MGST1 promoter has a known response element for oxidative stress and contains an antioxidant response element/electrophile response element (ARE/EpRE) site − 500 to the start site [28]. LTA4H is potentially a direct target for natural product-derived inhibitors [29]. There is suggestive evidence that plant quinones could directly block 5-LOX (ALOX5) enzyme activity, thereby blocking LTA4 and LTB4 synthesis [30], but it is not apparent how that direct inhibitory effect would affect the multiple ALOX transcripts modulated by Tr14.

### The Nrf2 system as a candidate mediator of phytochemical actions

Several members of the leukotriene pathway enzymes that were altered by Tr14, such as MGST3 and Fbp1 (Table 1), contain antioxidant response elements (ARE) that confer regulation by the transcription factor Nrf2 (NFE2L2) [28, 31, 32]. Leukotriene synthetic enzymes, such as MGST3 [33] and GGT1 [34], have been identified as Nrf2 target genes, but GGT1 is also responsive to TNF-A-induced NFkB and Sp1 [35]. Nrf2 is known to control multiple elements in the eicosanoid synthetic pathway, typically via the ARE, as in the case of thromboxane A2 synthase gene activation [36] and the prostaglandin reductase Ptgr1 [37]. Interestingly, early induction of Nrf2 by one of the rhomboid transcription factors has been shown to mediate rapid cutaneous wound healing [38].

### Phytochemicals and the Nrf2 pathway

Plant phytoestrogens and dietary polyphenols are known to regulate the Nrf2/Keap1 interaction in a manner that favors Nrf2 translocation to the nucleus, and Nrf2-dependent transcription of target genes [39, 40]. Likewise, the inhibition of neuroinflammation by nut bioflavonoids has been attributed to the induction of the Nrf2/ARE antioxidant system [41]. Interestingly, the anti-inflammatory mechanism of the marine natural product, honaucin A, has been attributed to activation of the Nrf2/Keap1 system via direct covalent modification of the sulfhydryl residues in Keap1 [42]. Likewise, plant phenylpropanoid glycosides have been shown to modulate Nrf2-dependent heme oxygenase 1 (Hmox1, HO1) transcription via regulating the balance of Nrf2 and Bach1 in human keratinocytes [43].

### The NFkB pathway

In addition to the Nrf2 pathway, one must consider the possibility that the NFkB pathway is altered by the phytochemical treatments. Certain plant-derived isoflavanones, such as sappanone A, exert anti-inflammatory effects through both the Nrf2 and NF-kB pathways [44]. Many of the pathway members, such as MGST3 are modulated by both Nrf2 and NFkB [35], which complicates the problem of dissecting the precise transcription factors involved.

### A “Phytocellular” theory: phytochemicals as modulators of immune cell recruitment

In addition to the direct pharmacological effects of phytochemicals on the transcriptional pathways of viable cells that remain in a wound, it is potentially important to consider whether phytochemicals could alter the types of cells that respond to the injury. This was suggested by relatively large changes in several transcripts that had known relationships to particular immune cell types, especially T cells and the Treg subset. As summarized in Fig. 7, phytochemicals include biologically active compounds, such as polyphenols, retinoids, flavonoids, genistein, and many others, that have documented effect on the recruitment and activity of immune cells [45, 46]. In addition to a direct effect on the activity of the infiltrating immune cells, it is essential to consider that these same agents can alter the types of cytokines that are produced by the surviving resident cells in the wound, and alter their production of chemotactic agents. As shown schematically in Fig. 7, the healing wound must be considered in the context of an interplay between a range of cell types that includes keratinocytes, fibroblasts, dermal plasmacytoid dendritic cells, mast cells, neutrophils, B cells, T cells, especially gamma/delta and Treg, and natural killer (NK) cells (see [47] for review).

There is extensive data to support the concept that this cellular interplay is inseparable from the inflammatory and resolving chemical signals. Broadly, pro-inflammatory components of wound repair must be tempered by pro-resolution factors, or the result can be a range of defects that ranges from chronic wounds, autoimmunity, hypertrophic scarring and keloid formation [48]. If, as the current data suggests, phytochemicals alter the type of cells that accumulate in the wound during repair, then there is a plausible explanation for their observed beneficial effects. In this ‘phytocellular’ framework, it is interesting to speculate that if Tr14 were to increase the timely infiltration of Treg cells, this could limit the pro-inflammatory effects of tissue damage. Future studies will require detailed RNA in situ hybridization and immunochemistry studies at selected time points to evaluate this hypothesis.

## Conclusions

- The biological effects of Tr14, a multicomponent drug, are largely different than diclofenac, and impact different subsystems in the COX/LOX pathways during wound repair.
- Diclofenac, but not Tr14, inhibits COX-2 mRNA induction by blocking downstream PGE2 production.
- Tr14, but not diclofenac, reduces mRNA levels in the leukotriene synthetic pathway, possibly by activating the Nrf2/KEAP2 pathway.
- Tr14 causes a transcriptomic signature that is consistent with alterations in the types of cells that are present in the wound, and appears most consistent with elevated Treg composition in Tr14-treated wounds.

## Methods

### Wound healing model

The overall experimental workflow and data analysis is shown in Fig. 1. Animal care and use was approved by the Nantong University Animal Care Committee and complied with all relevant guidelines for humane use. The ICR strain of mice in the age range of 4–6 weeks, approximately 20 g each, was used for the wound healing studies. The skin abrasion model is based closely on prior published work that documents the histological changes over time and in response to laser stimulation [49]. Under sedation (ketamine 100 mg/kg, xylazine 10 mg/kg IP), the mouse dorsal/scapular region was shaved and then a 1 cm^2^ area was wounded by rotary abrasion. The abrasion results in a partial-thickness wound with removal of the epidermis and part of the dermis, with a mild superficial bleeding response that quickly scabs, does not require analgesia post-operatively. In prior publications, the temporal association between the histological/cellular stage of repair and gene expression is reported in this same model [3].

To optimize its effect, Tr14 was introduced as subcutaneous injections of 0.1 ml (9.5 ml/ml) in the region around the wound, without sedation, with twice daily topical treatment (34 mg/ml) to the wound (TTI group) and compared to mice treated with saline injections (SI). Diclofenac was applied at clinically relevant doses of 2 mg/ml. The diclofenac group (DT) and its control group (PT) did not receive sc injections. A standardized 1 cm^2^ piece of affected skin was recovered by sacrifice, via ketamine/xylazine overdose and cervical dislocation, at specific times: 0, 12, 24, 36, 72, 96, 120, and 192 h after injury. Tissue was stored in RNAlater at − 80 °C until RNA was isolated as described below. Each time point of each treatment or control group included 7 mice.

### Isolation of Total RNA from tissues

Total RNA from tissue samples and cell lines was extracted using TRIzol (Invitrogen) using the manufacturer’s protocol. The sample was homogenized with a Tissue Tearer rotary blade device while suspended within an appropriate volume (10x sample volume) of TRIzol [50]. After 10 min at room temperature, the samples were centrifuged to remove insoluble material and the supernatant transferred to a fresh tube. Chloroform (0.2X TRIzol volume) was then added to the contents of the new tube and vigorously vortexed. Samples were then centrifuged for 15 min at 12,000 x g at 4 °C. The upper aqueous phase, which contains RNA, was then carefully transferred to a new tube. The addition of isopropyl alcohol (0.5X TRIzol volume) to the samples followed by a 10-min room temperature incubation and a subsequent 10-min centrifugation at 12,000 x g at 4 °C precipitated the RNA into a gel-like pellet at the bottom of the tube. After the removal of the supernatant, the pellet was washed twice in 1 ml of ice-cold 75% ethanol. The resulting pellet of RNA was then allowed to dry for 10 min and then resuspended in DEPC-treated water. The quantity of the resulting RNA was measured by the absorbance at 260 nm, and the relative contamination with proteins was measured by the ratio of optical density (OD) at 260/280 on a NanoDrop instrument (Thermo Scientific). A ratio greater than 1.8 is desirable.

### Depletion of genomic DNA (gDNA) and ribosomal RNA (rRNA)

In addition to messenger RNA (mRNA) and non-coding RNA (ncRNA), total RNA extracted from tissue samples contain large quantities of ribosomal RNA (rRNA), transfer RNA (tRNA) and residual amounts of genomic DNA (gDNA). The sequencing reads are used most efficiently if the rRNA and gDNA is depleted prior to sequencing.

#### DNAse treatment

Total nucleic acid from the TriZol step is first DNase-treated to remove any residual DNA. Approximately 40 μg of total RNA (with 20 μl 10x buffer, 4 μl DNase 1 (Ambion), 2 μl Rnase-out (Invitrogen) in a total volume of 200 ul) is incubated for 30 min at 37 °C. Samples are then cleaned using the RNeasy MinElute cleanup kit (Qiagen) following manufacturer’s protocol. In brief, 700 μl Buffer RLT and 500 μl 100% ethanol are added to the sample which is then added to a MinElute spin column. The columns are washed with 500 μl Buffer RPE followed by 500 μl 80% Ethanol. After an additional 2-min centrifugation to remove any residual ethanol, the sample is eluted in 14 μl DEPC treated water. The quality of RNA was then assessed using an Agilent 2100 Bioanalyzer and the RNA 6000 Nano Kit (Agilent) using manufacturer’s protocol and the sample was quantified using a Nanodrop, as described above.

#### rRNA depletion

Samples were depleted of ribosomal RNA through the use of the RiboMinus Eukaryote Kit for RNA-Seq (Invitrogen) following the manufacturer’s protocol. In brief, 10 μg of sample RNA (in a total of 10 ul) were incubated with 10 μl RiboMinus probe and 100 μl Hybridization Buffer for 5 min at 75 °C then allowed to slowly cool to 37 °C over the course of 30 min. The RiboMinus probe contains 5′ biotinylated Locked Nucleic Acid probes that are complementary to conserved eukaryotic 5S, 5.8S, 18S and 28S ribosomal sequences. Streptavidin-coated RiboMinus Magnetic Beads were washed once in water, resuspended in hybridization buffer, separated into two aliquots and kept at 37 °C until use. Sample was added to the prepared beads and incubated at 37 °C for 15 min with occasional agitation. The beads were placed on a magnetic separator and the supernatant containing the enriched RNA was removed and added to the second aliquot of beads and the protocol repeated. The final supernatant (~ 320 ul) was precipitated using 1 μl glycogen (Invitrogen), 30 μl 3 M sodium acetate and 75 μl 100% ethanol. The mixture was incubated at − 80 °C for 1 h and then centrifuged for 15 min at 12000 x g at 4 °C. The pellet was washed twice with cold 75% ethanol, air dried for 5 min and then resuspended in 30 μl DEPC-treated water. Quality of RNA was then re-assessed using an Agilent 2100 Bioanalyzer and their RNA 6000 Pico Kit (Agilent) using manufacturer’s protocol and the sample was quantified using a Nanodrop as per above.

### Transcriptome sequencing sample preparation

A published description of the single-molecule sequencing methodology for transcriptome quantification is described by Lipson et al. [51]. A brief summary follows.

#### Complementary DNA (cDNA) synthesis

Complementary DNA (cDNA) from rRNA-depleted RNA was prepared using the Superscript III cDNA synthesis kit (Invitrogen). In brief, 200 ng of RNA was resuspended in 17 μl nuclease-free water and heated at 95 °C for 5 min to fragment the RNA and thus improve the eventual evenness of coverage. Random hexamers (10 μl of 50 ng/ul) and dNTPs (2 μl of 10 mM mix) were added to the RNA and the mixture was heated to 65 °C for 5 min, and then placed on ice for 2 min. A mixture of 10X buffer (5 μl), 0.1 M DTT (1 μl) and 25 mM MgCl2 (10 ul) was added to the tube and the mixture was incubated at 15 °C for 20 min. A mixture of RnaseOut (2.5 ul) and SSIII reverse transcriptase (2.5 μl) was then added to the tube and the sample incubated (25 °C 10 min, 40 °C 40 min, 55 °C 50 min, 85 °C 5 min, 4 °C hold). The RNA remaining in the sample was then degraded by the addition of 1 μl RNase H and 1 μl RNase If (New England Biolabs, M0243L). The resulting cDNA was then purified by the serial use of two Performa Gel Filtration Columns (EdgeBio, 42,453). The concentration of the resulting cDNA was then quantified using the Nanodrop as previously described [51].

#### Poly a tailing

This step adds 3′ poly-A tails to the cDNA, which facilitates hybridization to the flow cell for sequencing. Using 100 ng of the prepared cDNA in 28 μl water, 5 μl Helicos PolyA Control Oligos are added and the mixture incubated at 95 °C for 5 min followed by a 2-min ice incubation. A mixture of CoCl (5 μl), 10X TdT buffer (5 μl) Helicos PolyA tailing dATP (5 μl) and Terminal Transferase (2 μl) was then added to the sample with thorough mixing. The samples were then incubated (42 °C, 60 min, 70 °C 10 min, 4 °C hold). The success of the polyA tailing was determined through the use of a 3730 DNA Analyzer (Applied Biosystems, 3730S) following manufacturer’s procedures. In brief, 1 μl of sample was added to 8.9 μl formamide and 0.1 μl GeneScan-120 LIZ Size Standard (Applied Biosystems, 4,324,287), and the samples were denatured at 95 °C for 2.5 min then cooled on ice and run on the machine [51].

#### 3′ blocking and tailing oligo removal

After addition of poly A tail, the 3′ tail of the cDNA sample is then blocked, and the tailing oligo is removed. The sample is denatured at 95 °C for 5 min followed by a 2-min incubation on ice. 0.4 μl biotinylated ddATP (Perkin Elmer) and 2 μl Terminal Transferase (New England Biolabs) are added and the samples incubated (37 °C 60 min, 70 °C 10 min). The sample is then digested with 1 μl USER enzyme (New England Biolabs) at 37 °C for 30 min [51].

#### Sample cleanup

The samples obtained from prior steps are then cleaned up using AMPure Beads (Beckman Coulter). The beads are allowed to equilibrate at room temperature for 30 min before use. Each probe sample is then brought up to a total volume of 60 μl and mixed with 72 μl AMPure beads and incubated at room temperature for 30 min with occasional mixing. Using a magnetic stand, the beads are then collected, and the beads are washed twice with 500 μl 70% ethanol. After the final wash the beads are allowed to dry for 5–10 min to remove all traces of ethanol. The beads are then suspended twice in 20 μl TE buffer and the supernatant containing the probes removed to a clean tube each time after 5 min on the magnetic stand. Samples are then precisely quantified using the OptiHyb procedure. In brief, oligo-dT coated mRNA Catcher Plus plates (Invitrogen) are used to collect replicates of 2 μl of sample in 48 μl hybridization buffer (1x SSC, 0.5% SDS). After a one-hour incubation at 37 °C the plates are washed 3x in Wash Buffer B (150 mM HEPES, 0.1% SDS, 1X SSC) then blocked for 15 min at room temperature in Blocking Buffer (0.1 M Tris pH 7.6, 0.15 M NaCl, 0.5% Casein). Streptavidin-HRP conjugate (Thermo Fisher) is added to Block buffer and the plates are incubated in that mix for 1 h at room temperature. After 3 washes in Wash Buffer 2 (0.1 M Tris pH 7.6, 0.15 M NaCl, 0.05% Tw20) the plates are dried and then incubated for 30 min in the dark with TMB Chromogenic Substrate (Sigma). Upon addition of 1 N HCl the samples are then measured at 450 nm on an EnVision Multilabel Reader (PerkinElmer). Concentrations were determined by comparison to a control dilution on the same plate.

#### Sample loading

After the measurements, the samples are loaded onto the flow cells. In brief, appropriate quantities of samples are diluted into hybridization buffer. The flow cells are then rehydrated for 3 h and brought to 55 °C. The flow cells are then equilibrated with loading buffer and then the samples are loaded and allowed to hybridize for 1 h to the flow cells, which are coated with oligo dT that hybridizes the polyA tails of the samples. The flow cells are then repeatedly washed to remove excess sample. A single fluorescent nucleotide is then added to each of the annealed probes on the flow cell so that, upon loading into the Heliscope, the location of each individual sample molecule can be determined. The flow cells are then loaded into the Heliscope and subsequent sequencing is automated [51].

### Sequencing operation and monitoring

Once the flow cells are loaded onto the SeqLL SMS, sequencing chemistry begins. Fluorescently labeled nucleotides are added one at a time to the flow cells. These nucleotides bind to complementary bases on the cDNA strand, whose 3′ Poly A tail is hybridized to the oligo dT probes in the flow cell. Unbound nucleotides are then washed away. Bound fluorescent nucleotides emit light under a laser beam, which is captured by a CCD camera. This determines which nucleotide is incorporated and the position in the template. The fluorescently labeled portion of the nucleotide is then cleaved off. The process is repeated for all four nucleotide bases and continued until a sequence read of desired read length is obtained. On average 35 nt. reads are generated with a throughput of 105 to 140 megabases per hour.

### Bioinformatics data analysis

#### Filtering and alignment

Each channel produces an average of 45 million total reads, which are then filtered as previously described, using the Helisphere software (v 1.2.740) to remove low complexity sequences and sequencing artifacts [52]. Low complexity sequences, such as poly A, and short reads under 25 nucleotides are removed by this process. Reads were aligned to mouse genome mm10 combined with SILVA ribosomal RNA reference sequences (LSUr123, SSUr123). To avoid ambiguity, we selected uniquely aligned reads only. The number of reads spanned the exon intervals of known transcripts were calculated using a custom perl script. The sum of exonic reads counts for each transcript is treated as raw Digital Gene Expression (raw DGE). Out of 138,930 transcripts we selected only one per gene using the following criteria: a) one transcript with maximum exon lengths was selected for each gene, b) the transcripts with names started from “Gm” and “Rik” were removed. At the end of the pipeline 25,482 genes/transcripts remained. With mapping data as input, transcript expression levels are calculated and presented in units of RPK10M (reads per thousand (K) nucleotides length of transcript, per 10 million reads captured per sample.) Previous studies confirm that quantitative expression levels generated by this process meet or exceed quality levels of microarray data, as measured by RT-PCR validation [53].

#### Differentially expressed genes (DEG)

We calculate fold change and *p*-value (probability to get that or better fold change by chance) for each of 138,930 transcripts when comparing between two different conditions (i.e. diclofenac vs topical placebo). Fold change is log2 scaled. To normalize raw DGE counts and calculate *p*-values, the DEseq2 Bioconductor software package (version 1.22.2) was employed [54]. An over-dispersed Poisson model was used to account for both biological and technical variability. To calculate p-values the Likelihood ratio test (LRT) option was used. It allows to identify any genes that show change in expression across the different time points. Benjamini-Hochberg method was used to correct the *p*-values for multiple testing. DEGs in the comparison of Diclofenac versus it’s control were defined through transcripts with adjusted p-value less than 10^− 6^. To define DEGs of Tr14 versus control the adjusted p-value cutoff 10^− 3^ was applied. The transcripts with names started from “Gm” and “Rik” were removed from DEGs list. In total 5615 transcripts (corresponding to 2218 genes) remained in the joint list of Diclofenac and Tr14 DEGs (Suppl. Table 1).

#### Transcriptome data analysis

Using data analysis generated above, several systems-level analyses were performed. To understand the correlations and patterns among different elements of the transcriptome, DEGs are mapped to their respective biological pathways and Gene Ontology (GO) categories [55]. Changes enriched in individual pathways or ontologies are tested for their statistical significance. GO categories that have a number of hits greater than expected by chance with adjusted p-value less than 0.05 are listed in the results. *P*-value is calculated using Hypergeometric model and adjusted for multiple testing using the Benjamini-Hochberg method.

## Supplementary Information

**Additional file 1: Supplementary Figs. 1–6.** KEGG analysis of the COX/LOX pathway at multiple time points.

**Additional file 2: Supplementary Table 1.** Comprehensive list of DEGs responding to diclofenac and Tr14 over time.

## Data Availability

The data sets generated and analyzed during the current study are available in NCBI Sort Read Archive (SRA) as BioProject PRJNA726431, (https://trace.ncbi.nlm.nih.gov/Traces/study/?acc=PRJNA726431).
