# Supplementary material for: RNAseq analysis of treatment-dependent signaling changes during inflammation in a mouse cutaneous wound healing model
Source: BMC Genomics. 2021 Nov 25;22:854. doi: 10.1186/s12864-021-08083-2 (PMC8614049; doi:10.1186/s12864-021-08083-2)
Supplement: Supplementary file 1 — Additional file 1: Supplementary Figs. 1–6. KEGG analysis of the COX/LOX pathway at multiple time points. [file 12864_2021_8083_MOESM1_ESM.pdf]

Supplementary Figures

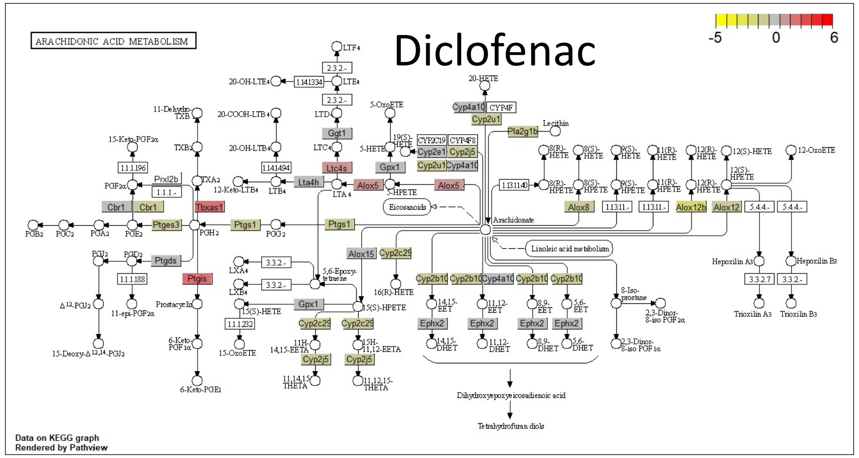

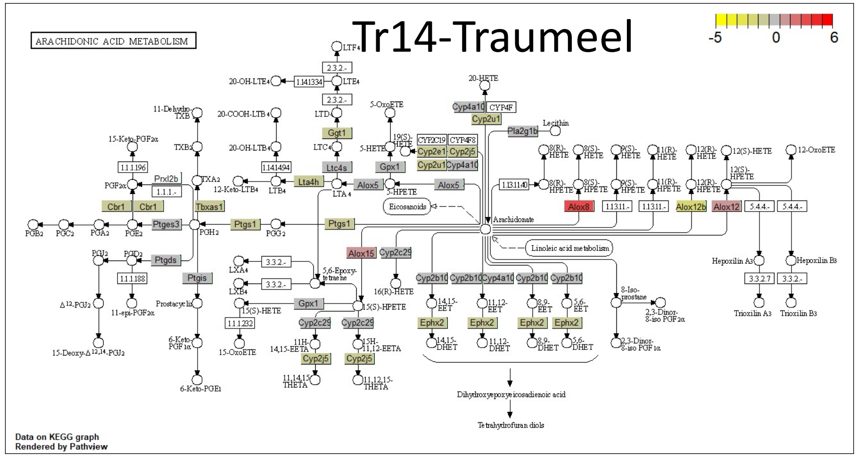

		12 HOUR	
**Gene**	**Transcript**	**Fold Change**	
**Symbol**	**Description**	**Tr14**	**Diclofenac**
Alox12	arachidonate 12-lipoxygenase	2.82	-1.54
Alox12b	arachidonate 12-lipoxygenase, 12B	-3.51	-3.15
Alox15	arachidonate 15-lipoxygenase	-1.43	3.93
Alox5	arachidonate 5-lipoxygenase	1.36	2.35
Alox8	arachidonate 8-lipoxygenase	11.73	-1.67
Cyp4f18	cytochrome P450, family 4f18	-1.12	-2.22
Pla2g12b	phospholipase A2, group XIIB	0.00	0.00
Pla2g1b	phospholipase A2, group IB, pancreas	2.27	0.00
Pla2g4d	phospholipase A2, group IVD	1.84	1.61
Ptgs1/COX1	prostaglandin synthase 1 (constitutive)	-1.47	1.15
Ptgs2/COX2	prostaglandin synthase 2 (inducible)	-1.26	-1.90
Tbxas1	Thromboxane A Synthase 1	-1.00	5.72

Supplemental Figure 1. Arachadonic Acid/prostaglandin/leukotriene pathway analysis of treatment-induced signaling changes at the 12H timepoint.

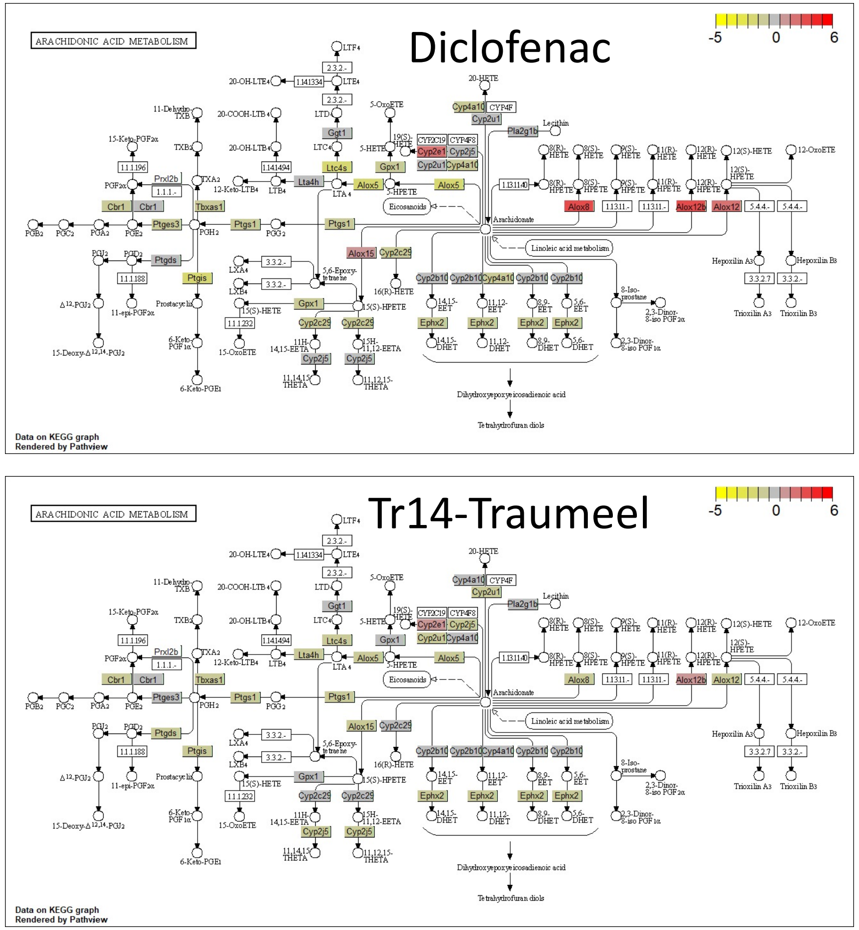

		36 HOUR	
Gene	Transcript	Fold Change	
Symbol	Description	Tr14	Diclofenac
Alox12	arachidonate 12-lipoxygenase	-1.29	3.40
Alox12b	arachidonate 12-lipoxygenase, 12B	2.06	10.99
Alox15	arachidonate 15-lipoxygenase	-1.04	-1.56
Alox5	arachidonate 5-lipoxygenase	-1.13	-2.30
Alox8	arachidonate 8-lipoxygenase	-1.93	8.71
Cyp4f18	cytochrome P450, family 4f18	-1.89	-1.57
Pla2g12b	phospholipase A2, group XIIB	1.18	-2.20
Pla2g1b	phospholipase A2, group IB, pancreas	0.00	-1.61
Pla2g4d	phospholipase A2, group IVD	2.46	5.58
Ptgs1/COX1	prostaglandin synthase 1 (constitutive)	1.08	-1.36
Ptgs2/COX2	prostaglandin synthase 2 (inducible)	-1.35	1.13
Tbxas1	Thromboxane A Synthase 1	-1.45	-1.28

Supplemental Figure 2. Arachadonic Acid/prostaglandin/leukotriene pathway analysis of treatment-induced signaling changes at the 36 H timepoint.

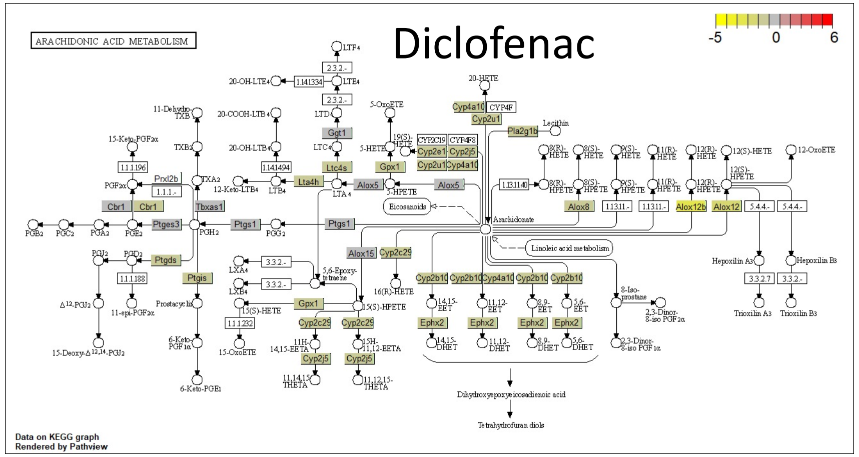

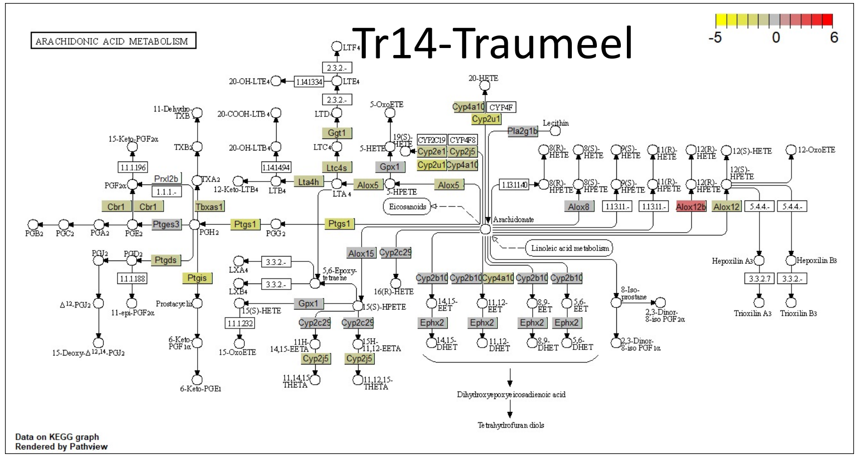

		72 HOUR	
Gene	Transcript	Fold Change	
Symbol	Description	Tr14	Diclofenac
Alox12	arachidonate 12-lipoxygenase	-1.59	-1.11
Alox12b	arachidonate 12-lipoxygenase, 12B	4.93	-4.73
Alox15	arachidonate 15-lipoxygenase	-1.24	1.37
Alox5	arachidonate 5-lipoxygenase	-1.08	1.47
Alox8	arachidonate 8-lipoxygenase	1.35	-1.23
Cyp4f18	cytochrome P450, family 4f18	-4.19	1.58
Pla2g12b	phospholipase A2, group XIIB	-2.71	-2.59
Pla2g1b	phospholipase A2, group IB, pancreas	-1.92	3.91
Pla2g4d	phospholipase A2, group IVD	1.79	-1.81
Ptgs1/COX1	prostaglandin synthase 1 (constitutive)	-1.19	-1.16
Ptgs2/COX2	prostaglandin synthase 2 (inducible)	-6.99	2.19
Tbxas1	Thromboxane A Synthase 1	-1.44	1.23

Supplemental Figure 3. Arachadonic Acid/prostaglandin/leukotriene pathway analysis of treatment-induced signaling changes at the 72H timepoint.

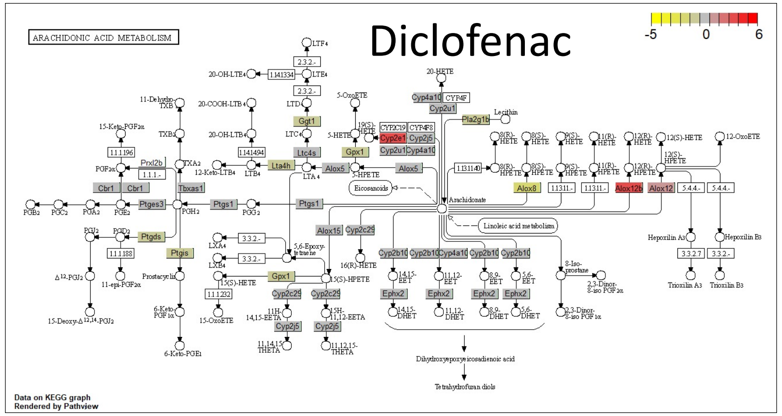

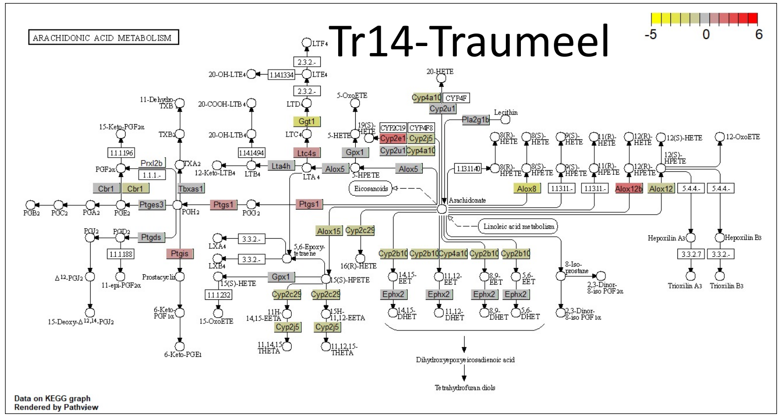

		96 HOUR	
**Gene**	**Transcript**	**Fold Change**	
**Symbol**	**Description**	**Tr14**	**Diclofenac**
Alox12	arachidonate 12-lipoxygenase	1.02	2.05
Alox12b	arachidonate 12-lipoxygenase, 12B	4.72	8.38
Alox15	arachidonate 15-lipoxygenase	1.44	3.18
Alox5	arachidonate 5-lipoxygenase	1.27	1.24
Alox8	arachidonate 8-lipoxygenase	-2.59	-2.54
Cyp4f18	cytochrome P450, family 4f18	2.03	2.34
Pla2g12b	phospholipase A2, group XIIB	3.22	-1.88
Pla2g1b	phospholipase A2, group IB, pancreas	3.08	-2.75
Pla2g4d	phospholipase A2, group IVD	3.52	3.05
Ptgs1/COX1	prostaglandin synthase 1 (constitutive)	1.65	1.50
Ptgs2/COX2	prostaglandin synthase 2 (inducible)	3.34	1.36
Tbxas1	Thromboxane A Synthase 1	1.93	1.23

Supplemental Figure 4. Arachadonic Acid/prostaglandin/leukotriene pathway analysis of treatment-induced signaling changes at the 96 H timepoint.

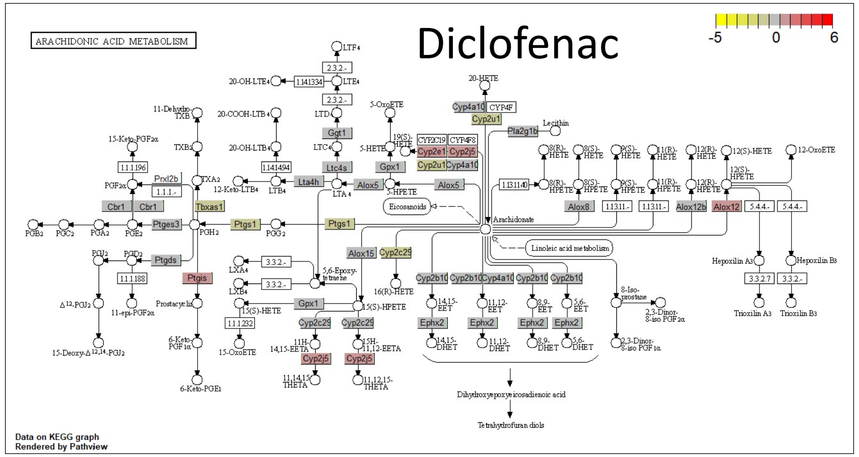

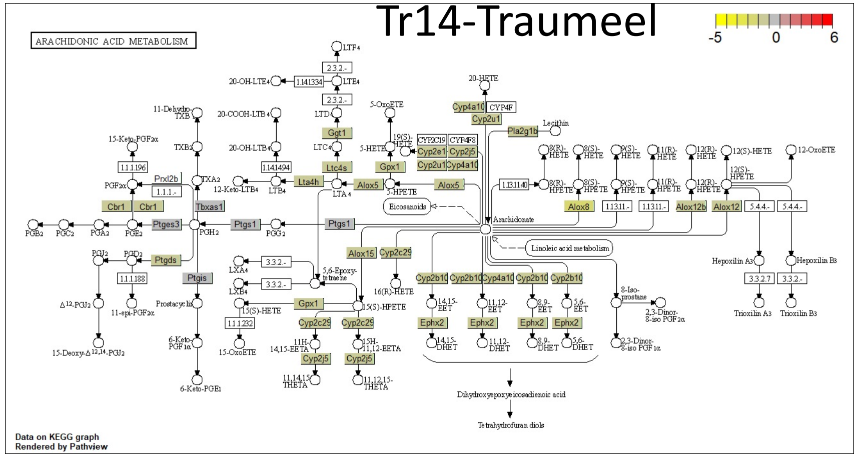

Gene Symbol	Transcript Description	120 HOUR Fold Change Tr14	Diclofenac
Alox12	arachidonate 12-lipoxygenase	-2.06	1.01
Alox12b	arachidonate 12-lipoxygenase, 12B	-1.18	1.51
Alox15	arachidonate 15-lipoxygenase	-1.09	1.79
Alox5	arachidonate 5-lipoxygenase	-1.56	1.26
Alox8	arachidonate 8-lipoxygenase	-2.75	1.59
Cyp4f18	cytochrome P450, family 4f18	1.88	-1.93
Pla2g12b	phospholipase A2, group XIIB	0.00	0.00
Pla2g1b	phospholipase A2, group IB, pancreas	-1.23	1.88
Pla2g4d	phospholipase A2, group IVD	-1.51	1.11
Ptgs1/COX1	prostaglandin synthase 1 (constitutive)	-1.08	1.72
Ptgs2/COX2	prostaglandin synthase 2 (inducible)	2.87	-2.50
Tbxas1	Thromboxane A Synthase 1	1.51	-1.36

Supplemental Figure 5. Arachadonic Acid/prostaglandin/leukotriene pathway analysis of treatment-induced signaling changes at the 120 H timepoint.

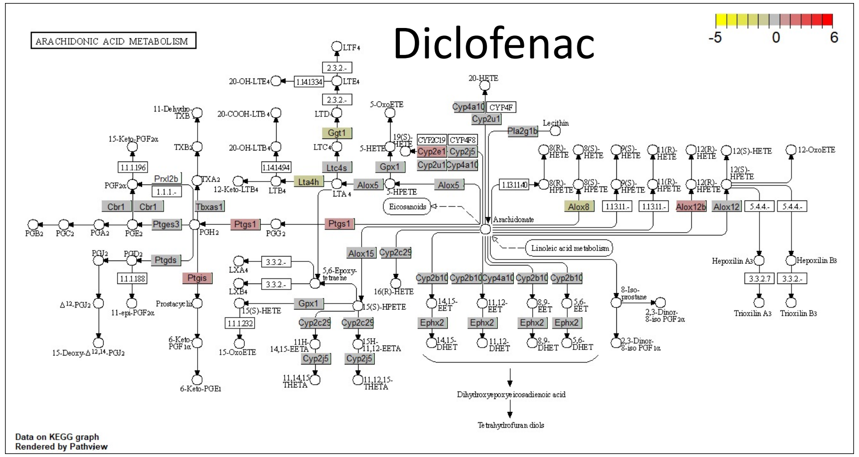

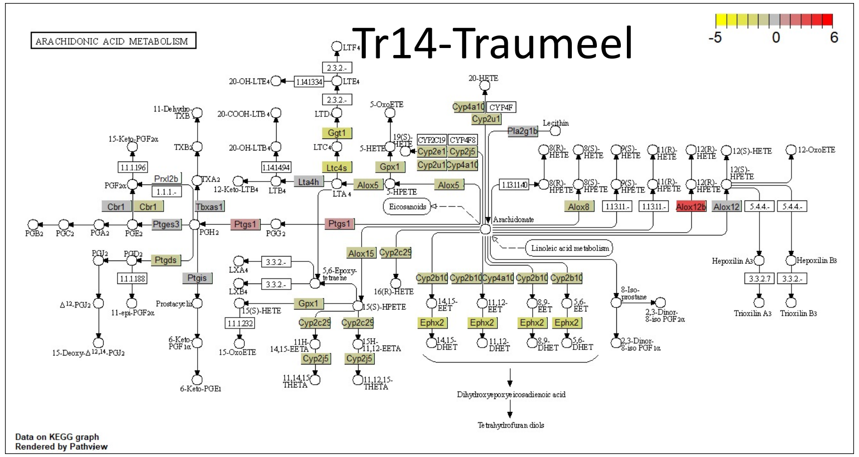

		192 HOUR	
**Gene**	**Transcript**	**Fold Change**	
**Symbol**	**Description**	**Tr14**	**Diclofenac**
Alox12	arachidonate 12-lipoxygenase	1.80	1.51
Alox12b	arachidonate 12-lipoxygenase, 12B	13.37	2.27
Alox15	arachidonate 15-lipoxygenase	-1.03	2.24
Alox5	arachidonate 5-lipoxygenase	-1.80	1.23
Alox8	arachidonate 8-lipoxygenase	-1.09	-1.95
Cyp4f18	cytochrome P450, family 4f18	1.31	2.03
Pla2g12b	phospholipase A2, group XIIB	0.00	1.99
Pla2g1b	phospholipase A2, group IB, pancreas	-1.04	-1.71
Pla2g4d	phospholipase A2, group IVD	13.58	5.32
Ptgs1/COX1	prostaglandin synthase 1 (constitutive)	1.52	1.95
Ptgs2/COX2	prostaglandin synthase 2 (inducible)	2.76	3.21
Tbxas1	Thromboxane A Synthase 1	1.06	1.04

Supplemental Figure 6. Arachadonic Acid/prostaglandin/leukotriene pathway analysis of treatment-induced signaling changes at the 192 H timepoint.
